# Application of Plasma Exchange in Steroid-Responsive Encephalopathy

**DOI:** 10.3389/fimmu.2019.00324

**Published:** 2019-02-27

**Authors:** Yuting Jiang, Xin Tian, Yixue Gu, Feng Li, Xuefeng Wang

**Affiliations:** ^1^Chongqing Key Laboratory of Neurology, Department of Neurology, the First Affiliated Hospital of Chongqing Medical University, Chongqing, China; ^2^Center of Epilepsy, Beijing Institute for Brain Disorders, Beijing, China

**Keywords:** plasma exchange, steroid, clinical practice, course, onset time, side effects

## Abstract

Plasma exchange has been widely used in autoimmune neurological diseases and is the standard treatment for myasthenia gravis crisis and Guillain-Barre syndrome. A growing body of research suggests that, in the clinical application of steroid-responsive encephalopathy, such as for Hashimoto's encephalopathy, limbic encephalitis, systemic lupus erythematosus encephalopathy, ANCA-associated vasculitis encephalopathy, and acute disseminated encephalomyelitis, plasma exchange is a safe, and effective option when steroids or other immunosuppressive therapies are ineffective in the short term or when contraindications are present. Additionally, plasma exchange can also be used alone or in combination with steroids, immunoglobulins, or other immunosuppressive agents to treat steroid-responsive encephalopathy. This paper reviews the clinical application of plasma exchange in steroid-responsive encephalopathy, including its indications, onset time, course, curative effects, and side effects.

## Introduction

Plasma exchange is also known as therapeutic plasma exchange (1). The seventh special issue of the Therapeutic Apheresis in Clinical Practice treatment guidelines, published in 2016 by the American Society for Apheresis, defines plasma exchange as a therapeutic procedure in which the patient's blood is separated into plasma and other blood components by medical devices, and then the plasma is removed and replaced by a replacement solution such as a colloidal solution (albumin and/or plasma) or a combination of crystal/colloidal solutions, thus eliminating or reducing unwanted substances (2). Castillo et al. (3) considered encephalopathy to be accompanied by cognitive impairment and one or more of the following: (i) neuropsychiatric symptoms (hallucinations or delusions and paranoia); (ii) myoclonus; (iii) seizure; and/or (iv) focal neurologic deficits. Steroid responsiveness refers to the complete or nearly complete return to normal neurological baseline status after steroid treatment, while steroid unresponsiveness refers to lack of improvement after at least 4–6 weeks of a sufficient dose of steroids (4). In this paper, steroid-responsive encephalopathy is a general term used to describe diseases characterized by diffuse brain injury and their responsiveness to steroids. These diseases include Hashimoto's encephalopathy, limbic encephalitis, systemic lupus erythematosus encephalopathy, ANCA-associated vasculitis encephalopathy, and acute disseminated encephalomyelitis. Plasma exchange is a rapid-onset, safe, and effective option for patients with steroid-responsive encephalopathy who fail to respond to steroids in the short term or for patients who are unable to tolerate the side effects of steroid therapies. It can also be used as an initial treatment (see Table 1).

**Table 1:** Case reports of plasma exchange for steroid-responsive encephalopathy.

**References**	**Diagnosis**	**Number**	**Clinical feature**	**Failed treatments**	**Process of plasma exchange**	**Side effects**	**Therapeutic effect**
Nagpal and Pande (5)	Hashimoto's encephalopathy	One	52-year-old female, aphasia, memory loss, and tremor	Corticosteroids, thyroxine	Three plasma exchange procecures; the volume of Plasma exchanged per procedure was 1.8 L.	Not mentioned	After the third plasma exchange, all neuropsychiatric symptoms improved.
Gul et al. (6)	Hashimoto's encephalopathy	Two	Case 1: 8-year-old female, status epilepticus	Antiepileptic drugs, immunoglobulin, steroids	Five sessions	Not mentioned	Status epilepticus was controlled after plasma exchange.
			Case 2: 17-year-old female, status epilepticus	Immunoglobulin, Antiepileptic drugs, steroids	Five sessions	Not mentioned	After plasma exchange, epilepsy was controlled without recurrence.
Simmons and Staley (7)	Hashimoto's encephalopathy	Two	Case 1: 55-year-old female, gait ataxia, memory loss, mood change	Mycophenolate, mofetil	Five sessions; the volume of plasma exchanged per session was one times the plasma volume.	Slight citrate toxicity	Three months after plasma exchange, the patient's gait ataxia improved without obvious changes in daily activities.
			Case 2: 49-year-old female, insomnia, altered state of consciousness, mental symptoms	Steroids, immunoglobulin	Two course courses no. 1 (one times the plasma volume/session for five sessions), the second and third course were all (one times the plasma volume/session for three sessions	Transient hypotension, headache	After the first plasma exchange, orientation and daily activity improved, and the anti-thyroid peroxidase antibody was reduced from >1,000 units before plasma exchange to 286 units. The anti-thyroid peroxidase antibody was 538 units at 1 month after the first course of plasma exchange, the psychiatric symptoms were improved after the second and third courses, and the anti-thyroid peroxidase antibody was < 63 units after the third course of plasma exchange.
Endres et al. (8)	Hashimoto's encephalopathy	One	55-year-old female, rapid progressive dementia, stupor, silence	Antidepressants, antipsychotics, steroids	Five sessions	Not mentioned	After plasma exchange, all symptoms were significantly improved, but verbal fluency was still lacking.
Tran et al. (9)	Hashimoto's encephalopathy	One	72-year-old female, cerebellar ataxia, cognitive dysfunction	Steroids, mycophenolate, immunoglobulin	Induction period: Two sessions/week for 6 weeks and then one procedure/week (1.5 times the plasma volume for 6 weeks, followed by 2 sessions/week (one times the plasma volume) and then 1 procedure/week. After 9.5 months, readjust to two sessions/week	Citrate toxicity somnolence lower limb spasm	Clinical symptoms were alleviated after the induction of plasma exchange, the patient could walk normally, and memory was restored. Anti-thyroid peroxidase antibody levels decreased, but when the plasma exchange sessions were reduced to one procedure/week, and the patient experienced recurrence. Therefore, plasma exchange was administered at the rate of 2 sessions/week, and the patient's gait was improved (stable and lively).
Batra et al. (10)	Anti-NMDA receptor limbic encephalitis	One	34-year-old female, abnormal mental behavior,	Acyclovir, immunoglobulin	1.2 times the plasma volume/ procedure for five sessions, once every other day, with 5% albumin as the replacement fluid	Not mentioned	Symptoms improved significantly after 1 month, and antibody tests were negative. Symptoms were significantly relieved.
Mazzi et al. (11)	Nonparaneoplastic limbic encephalitis associated with the anti-GAD antibody	One	21-year-old female, intractable epilepsy, memory loss	Antiepileptics, steroids, immunoglobulin	Sixteen sessions with 1.4 times the plasma volume replaced during each procedure	Not mentioned	Seizures were significantly reduced after the third plasma exchange session.
Martin et al. (12)	Anti-VGKC antibody-positive limbic encephalitis	One	69-year-old male, memory loss, Abnormal mental behavior	Steroids, immunoglobulin, mycophenolate, mofetil	One time the plasma volume/session (2 sessions/week) for 6 weeks	Not mentioned	Anti-VGKC antibody levels decreased, and clinical symptoms were well controlled.
Özkale et al. (13)	Two cases of anti-NMDA receptor encephalitis One case of serologic negative Autoimmune Limbic encephalitis One case of paraneoplastic limbic Encephalitis	Four	Headache, Status epilepticus psychiatric symptom	Steroid, immunoglobulin	Six sessions on average	Not mentioned	The neurological symptoms of all patients improved after plasma exchange.
Hussein et al. (14)	Systemic lupus erythematosus encephalopathy	One	17-year-old female headache, double vision, facial paralysis	Cyclosporin	Six sessions	Not mentioned	Cyclophosphamide and methylprednisolone (1 g/d for 3 days) combined with six plasma exchange resulted in significant improvement in clinical symptoms.
Bartolucci et al. (15)	Systemic lupus erythematosus encephalopathy	Ten	Average age: 30 Female: 8, male: 2 psychiatric symptoms, aseptic meningitis, cognitive decline	Not mentioned	Three sessions/week for three weeks and then two or three sessions/week, according to the clinical outcome	Not mentioned	After plasma exchange, 54% (7/13) had complete remission, and 46% (6/13) had partial remission. The European consensus lupus activity score (ECLAM) and systematic lupus activity index (SLEDAI) decreased to 1.2 and 3.9 respectively from 6.9 and 30.7.Disappeared upon head MRI examination, and consciousness level and mental state began to recover. The PR3-ANCA antibody decreased from 164 U/ml to 15.7 U/ml.
Shinozaki et al. (16)	Acute disseminated encephalomyelitis	One	Headache, confused and delirious state, no eyelash, corneal or light reflexes	Intravenous methylprednisolone (1,000 mg per day for 5 days)	Plasma exchange (PE) plus continuous hemodiafiltration using 1.5 times the circulating plasma volume, daily PE with CHDF for 3 days	Not mentioned	On the day after initiation of SPE + CHDF, the patient's corneal and bilateral light reflexes were dramatically recovered.
Balestri et al. (17)	Acute disseminated encephalomyelitis	One	An eight-year-old female, comatose and generalized tonic-clonic seizures, unable to raise her head, walk, or speak, and facial paresis	Methylprednisolone, immunoglobulin, and Interferon beta-1b	Six courses with 1,400 cc of plasma exchanged once every 2 days)	Not mentioned	Dramatic recovery was observed over the next 2 weeks, and the patient was able to walk, speak, and raise her left forearm.

## History of Plasma Exchange

Plasma exchange dates back to 1914. Able et al. (18) described the separation of cell components and plasma from the blood of dogs with uremia. The separated components were mixed with replacement solution and then returned to the subject. Regular plasma exchange began to be used in humans in 1952. Researchers found that repeated plasma exchange reduced the amount of pathological proteins in patients with multiple myeloma (19). In 1960, Schwab and Fahey (20) reported that plasma exchange in Waldenstrom's macroglobulin and hyperviscosity syndrome achieved good therapeutic effects. Therefore, plasma exchange became the standard treatment for Waldenstrom's macroglobulin. In the 1980s, studies reported that plasma exchange was an effective treatment for systemic lupus erythematosus encephalopathy and acute disseminated encephalomyelitis; thereafter, plasma exchange began to be used as a treatment for steroid-responsive encephalopathy (4, 21).

## Unknown Mechanisms of Plasma Exchange or Potentially Involved Mechanisms Under Exploration

### Clearing Pathogenic Antibodies From Plasma

The mechanism of plasma exchange for treating systemic lupus erythematosus encephalopathy is the rapid removal of pathogenic autoantibodies such as anti-nuclear antibodies from the blood (22). Anti-neutrophil cytoplasmic antibodies (ANCAs) play an important role in the pathogenesis of ANCA-related vasculitis encephalopathy, and the clearance of pathogenic antibodies from blood by plasma exchange can improve the therapeutic effects (2). Plasma exchange can also effectively remove pathogenic antibodies and can be combined with immunotherapy to suppress the production of autoantibodies and effectively treat limbic encephalitis (23). The mechanism of plasma exchange for treating acute disseminated encephalomyelitis is the removal of autoantibodies (antibodies against myelin oligodendrocyte glycoprotein) as well as complement components and cytokines (4).

### Increasing the Susceptibility of Antibody-Producing Cells to Immunosuppressant and Chemotherapeutic Drugs

Plasma exchange can also induce proliferation of antibody-producing cells and increase the synthetic ability of antibodies as well as the susceptibility of antibody-producing cells to immunosuppressive or chemotherapy drugs (23). Studies have reported that plasma exchange can increase the synthetic activity of B cells and increase the susceptibility of antibody-producing cells to immunosuppressive agents (24).

### Removing Immune Complexes From Plasma and Enhancing the Function of Macrophages and Monocytes

Plasma exchange can not only directly promote the removal of immune complexes from patients with systemic lupus erythematosus (22) but also upregulate red blood cell (RBC) complement receptors and increase the binding of RBC and immune complexes to remove immune complexes from the circulation. Steven et al. (25) studied the effect of plasma exchange on monocyte function and found that monocytes significantly increased their bactericidal effect by increasing the level of proteolytic enzymes in immune-complex-mediated diseases.

### Removing Pathogenic Cytokines and Adhesion Molecules From Plasma

The concentration of soluble adhesion molecules ICAM-1 and VCAM-1 may be decreased after plasma exchange in patients with ANCA-associated vasculitis (26). Yeh et al. (27) found that double-filtration plasmapheresis can effectively remove IL-2, IL-4, IL-5, tumor necrosis factor alpha, and interferon gamma from the serum of patients.

## Clinical Application of Plasma Exchange in Steroid-Responsive Encephalopathy

### Indications of Plasma Exchange for the Treatment of Steroid-Responsive Encephalopathy

Therapeutic plasma exchange is an established treatment method for known or suspected immune-mediated diseases (28). In 2016, the American Society for Apheresis published treatment guidelines for plasma exchange based on evidence-based medical research and proposed that Hashimoto's encephalopathy is a category II indication for plasma exchange and that the recommended level is 2C. Anti-N-methyl-D-aspartate receptor encephalitis is a category I indication, and the recommended level is 1C. Plasma exchange for the treatment of severe systemic lupus erythematosus, including systemic lupus erythematosus encephalopathy, is a category II indication, and the recommended level is 2C (2). Plasma exchange for the treatment of acute disseminated encephalomyelitis is a class II indication, and the recommended level is 2C (2) (See Tables 2, 3).

**Table 2:** Category of recommendation for plasma exchange (2).

**Category for plasma exchange**	**Detailed description**
I	Plasma exchange is used as first-line treatment alone or in conjunction with other treatments.
II	Plasma exchange is used as second-line treatment alone or in conjunction with other treatments.
III	The optimal role of plasma exchange has not been determined, and decisions should be personalized.
IV	Published evidence confirms or suggests that plasma exchange is ineffective or even harmful and that IRB approval is required if it is to be used in these circumstances.

**Table 3:** Recommended levels for plasma exchange (2, 29).

**Recommendation**	**Grade 1A**	**Grade 1B**	**Grade 1C**	**Grade 2A**	**Grade 2B**	**Grade 2C**
The quality of evidence	RCT without significant limitation or overwhelming evidence from observational studies.	RCT has important limitations (inconsistent results, methodological defects) or exceptionally strong evidence from observational studies.	Case series or observational studies.	RCT without significant limitation or overwhelming evidence from observational studies.	RCT has important limitations (inconsistent results, methodological defects) or exceptionally strong evidence from observational studies.	Case series or observational studies.
Detailed description	Strong recommendation, high-quality evidence.	Strong recommendation, moderate-quality evidence.	Strong recommendation, low-quality, or very low-quality evidence.	Weak recommendation, high-quality evidence.	Weak recommendation, moderate-quality evidence.	Weak recommendation, low-quality, or very low-quality evidence.

### Volume, Interval Time, and Frequency of Plasma Exchange for the Treatment of Steroid-Responsive Encephalopathy

The efficacy of plasma exchange is often related to the volume of plasma exchanged, which is dependent on the estimated plasma volume of the patient. The formula for estimating the plasma volume of the patient uses the patient's weight and hematocrit: EPV=[0.065 × wt (kg)] × [1-Hct]. This formula provides a reliable prediction of the therapeutic effect in clinical applications. In general, macromolecular substances (immune globulin, lipoprotein cholesterol, cold globulin, etc.) inside and outside of blood vessels become slowly redistributed, achieving a gradual balance. Thus, clearance during a single treatment is limited. One is the concentration of substances in the blood vessels, while the other is the volume of plasma exchanged. Based on these two factors, the percentage of the decrease in pathogenic substances after treatment compared with the pretreatment level can be determined as follows: *X*_1_ = *X*_0*e*_-*V*_e_ /EPV, where *X*_1_ is the final plasma concentration, *X*_0*e*_ is the initial plasma concentration, *V*_e_ is the volume of plasma exchange, and EPV is the estimated plasma volume of patients. If the volume of plasma exchange is equal to the patient's EPV, pretreatment values will drop by 63%, and if the volume of plasma exchange is equal to 1.4 times the EPV, pretreatment values will drop by 75%. However, in the process of a single exchange, the volume of plasma exchanged is further increased. As a result, the pretreatment level decreases less, and thus, the exchange volume would increase, subsequently increasing the duration of treatment and associated costs. For most indications of plasma exchange (including Hashimoto's encephalopathy, limbic encephalitis, systemic lupus erythematosus encephalopathy, ANCA-associated vasculitis encephalopathy, acute disseminated encephalomyelitis, etc.), the volume of plasma exchanged per treatment is 1–1.5 times the plasma volume (30). For a single plasma exchange treatment, this volume will not cause reductions in the overall load of the serum levels caused by partial rebound. Several consecutive plasma exchange sessions, separated by 24–48 h, can remove a substantial percentage of the total body burden. In general, if the rate of production is moderate, then at least five sessions within 7–10 days are required to remove 90% of the patient's initial overall load, and additional sessions will be needed if the production is rapid (30).

### Curative Effects

Cook et al. (31) retrospectively analyzed plasma exchange for the treatment of 10 Hashimoto's encephalopathy cases and showed that 90% of the symptoms of Hashimoto's encephalopathy significantly improved after plasma exchange. Neuwelt (32) reported the use of plasma exchange in eight systemic lupus erythematosus encephalopathy patients who failed to respond to cyclophosphamide, among whom six were completely relieved of their clinical symptoms. In 2010, a non-blinded prospective study by Wong et al. (33) included nine cases of limbic encephalitis with positive anti-VGKC antibody, and each patient underwent five plasma exchange sessions combined with steroid and immunoglobulin treatment. After treatment, the VGKC antibody titer of all patients returned to normal within 1–4 months. After 1–3 months, clinical and cognitive tests showed that memory function had improved. After 6–9 months, the swelling subsided, and the signal was recovered on brain MRI.

### Adverse Reactions

Plasma exchange is a relatively safe treatment, mostly with reports of only mild side effects, of which the most common are hypotension, hypocalcemia, urticaria, bleeding (due to loss of platelets or clotting factors), and arrhythmia. These adverse reactions are mainly related to anticoagulants, the replacement fluid used, and central venous catheterization. The incidence of hypocalcemia is 1.5–9% and is related to citrate. The main symptoms include paresthesia, muscle spasm, and arrhythmia. In addition, acid-base imbalance can be induced by citrate. The use of albumin as a replacement fluid may lead to the consumption of clotting factors and immunoglobulin and thus increase the risk of bleeding and infection. Fresh frozen plasma used as a replacement solution may cause HIV and hepatitis virus infection (34). Adverse reactions associated with central venous catheterization include infection, sepsis, thrombosis, and pneumothorax. Hemolysis and hypotension may occur, but the incidence of serious side effects such as severe hypotension, acute pulmonary edema, myocardial infarction, and death is 1.6–22% (35). In 2007, the world plasma exchange registry reported that the incidence of side effects from plasma exchange was 5.7% and that no death occurred in 838 patients who underwent plasma exchange; a plasma exchange team in Canada analyzed 91,000 sessions of plasma exchange and found that the incidence of serious side effects caused by plasma exchange was 0.4%. In addition, blood transfusion-related side effects are more common when plasma is used as the replacement fluid (36). Basic-Jukic et al. (34) studied the side effects of plasma exchange in the treatment of neurological diseases, including 152 patients from January 1982 to December 2003, with a total of 4,857 plasma exchanges performed. The incidence of side effects was 4.74% (231/4857), and the side effects were mostly mild to moderate. In summary, a few studies have reported on the side effects of plasma exchange for steroid-responsive encephalopathy, the results indicate that plasma exchange may be a safe treatment for steroid-responsive encephalopathy.

## Application of Plasma Exchange in Different Types of Steroid-Responsive Encephalopathy

### Application of Plasma Exchange in Hashimoto's Encephalopathy

Hashimoto's encephalopathy (also known as autoimmune thyroiditis-related steroid-responsive encephalopathy) was first reported by the British scholar Brain in 1966. Hashimoto's encephalopathy is related to Hashimoto's thyroiditis, as anti-thyroid antibodies were found in serum. The patients' thyroid function can be classified as normal, hypothyroidism or hyperthyroidism (37, 38). The clinical manifestations mainly include two types: vasculitis type, mainly including recurrent stroke-like episodes, seizures, and mental abnormality, and diffuse progressive type, which manifests as cognitive dysfunction (including memory and language dysfunction) dementia, behavior change, confusion, mental derangement, and coma (5). The most common clinical manifestations are seizures, followed by psychiatric symptoms (39). Elevated levels of anti-thyroid peroxidase antibodies (anti-TPOAb) and/or anti-thyroglobulin antibodies (anti-TgAb) are important laboratory characteristics for the diagnosis of Hashimoto's encephalopathy; elevated anti-thyroid peroxidase antibody levels are most common and are observed in 86% of patients with Hashimoto's encephalopathy, while 48% of the patients with Hashimoto's encephalopathy have elevated anti-thyroglobulin antibody levels (6). Although the pathophysiological mechanism of Hashimoto's encephalopathy is still not clear, high concentrations of anti-thyroid antibodies and effective treatment with immunosuppressive agents both support the important role of autoimmune mechanisms in Hashimoto's encephalopathy (40), which is the theoretical basis of plasma exchange in the treatment of Hashimoto's encephalopathy. Although steroids are the first-line treatment for Hashimoto's encephalopathy (41), no randomized controlled trials have been performed, so the optimal dose and duration of steroids remain unclear. Steroid responsiveness is determined by the dose and administration method. Usually, intravenous methylprednisone (500–1,000 mg/d) is administered for 3–7 days, followed by oral prednisone 1–2 mg/kg/d for 6–8 weeks, and in most cases, clinical improvement is observed within the first 4–6 weeks of treatment (4). When patients are unable to tolerate the side effects of steroid or have no response to steroids in the short term, plasma exchange can be performed to improve treatment efficacy. Moreover, a few reports have demonstrated that plasma exchange can be used for the initial treatment (39).

#### History of Plasma Exchange as a Treatment for Hashimoto's Encephalopathy

In 2001, Boers and Colebatch (42) was the first to report that plasma exchange could effectively treat Hashimoto's encephalopathy that failed to respond to corticosteroids. The author reported a 47-year-old Uruguayan man who was treated for upper limb postural tremor and gait disorder. During hospitalization, the patient developed seizures, short-term memory impairment, visual hallucination, auditory hallucinations, and paranoid delusions. Electroencephalogram (EEG) showed diffuse slow wave activity but no epileptic discharge. Cerebrospinal fluid showed increased pressure and protein (1.06 g/l), but other cerebrospinal fluid examinations (including polymerase chain reaction of herpes simplex virus), brain magnetic resonance plain scan, and enhancement showed no abnormalities. Examinations for thyroid stimulating hormone (TSH) were normal, but the levels of microsomal antibodies and anti-thyroglobulin antibodies significantly increased, so the diagnosis of Hashimoto's encephalopathy was established after ruling out other causes. Intravenous methylprednisolone was initiated, but 4 weeks later, the patient still experienced tremor and difficulty eating and dressing himself, so four plasma exchange sessions were performed with the exchange of 1.5–2 times the estimated plasma volume per session. After the first plasma exchange, the patient's condition improved, and after the fourth plasma exchange, the patient was able to dress, eat, talk, and work independently; in addition, his antibody levels decreased. Afterward, the patient experienced two relapses, and the symptoms were relieved after plasma exchange. Multiple studies have subsequently supported this treatment (5, 6, 40, 43–45). Nieuwenhuis et al. (45) reported a 48-year-old patient with subacute Hashimoto's encephalopathy, and the main manifestations were rapid progressive dementia, visual hallucinations, and myoclonus. Plasma exchange was used as the initial treatment of Hashimoto's encephalopathy, and clinical symptoms relieved after the first plasma exchange.

#### Onset Time of Plasma Exchange for Hashimoto's Encephalopathy

Most of the cases in which plasma exchange used to treat Hashimoto's encephalopathy showed positive effects after the first plasma exchange. Clinical symptoms of Hashimoto's encephalopathy were found to be improved after the first plasma exchange in a report by Boers and Colebatch (42). In addition, Bektas et al. (43) reported one Hashimoto's encephalopathy in which the patient's status epilepticus was controlled after the first plasma exchange.

#### The Course of Plasma Exchange for Hashimoto's Encephalopathy

Most studies on plasma exchange for Hashimoto's encephalopathy lack a specific description of the number of plasma exchange sessions used. Nieuwenhuis et al. (45) used three sessions plasma exchange to treat Hashimoto's encephalopathy successfully. Bektas et al. (43) used nine sessions plasma exchange, while Nagpal and Pande (5) and Gul Mert et al. (6) reports that the number of plasma exchange sessions for the treatment of Hashimoto's encephalopathy should be five. These results are consistent with the American Society for Apheresis, which recommends a total of 3–9 sessions plasma exchange for Hashimoto's encephalopathy, with the most common number of plasma exchange being five and exchanges being performed once every other day. The volume of plasma exchanged per treatment should be 1–1.5 times the estimated plasma volume, and albumin should be used as the replacement solution (2, 5, 31, 45).

#### Clinical Practice of Plasma Exchange for Hashimoto's Encephalopathy

Hussain et al. (44) reported a case of 54-year-old woman with hypothyroidism who presented with progressive cognitive impairment, gait disturbance, and seizures; based on an anti-thyroid microsomal antibody titer of 1:1,600 and the presence of head abnormalities on MRI without other reasons for cognitive impairment, a definite diagnosis of Hashimoto's encephalopathy was made. The patient began oral prednisone at a dose of 60 mg/d, and her cognitive function, apraxia, and gait disorder improved, but memory impairment remained. Because the patient could not tolerate the side effects of prednisone, the dose was gradually reduced to 15 mg/d. As the patient's cognitive function and gait disorder worsened with the reduction in the dose of prednisone, plasma exchange was performed, with five sessions per course and two courses of plasma exchange at intervals of 5 months. Four weeks after the first course of treatment, the patient's cognitive function markedly improved, and anti-thyroid microsomal antibody levels were reduced to 1:400. Cognitive function began to decline a few months later, but after the patient underwent the second course of plasma exchange, cognitive function continuously improved. Pari et al. (40) reported a 19-year-old girl who had been in good health but experienced a seizure. One month later, she had difficulty finding words and understanding language, along with symptoms of confusion, and disorientation. Electroencephalogram showed a non-convulsive status epilepticus, with a slightly elevated number of cells in CSF, with normal glucose and protein levels. PCR analysis of herpes simplex virus, adenovirus and enterovirus in cerebrospinal fluid were all negative. Brain MRI was normal. An 18F-FDG PET on the left temporal lobe, insula, temporoparietal junction, the right side of the parietal lobe metabolism reduced, diagnosis of Hashimoto's encephalopathy, intravenous methylprednisolone 1 g/d for 8 days, but no obvious improvement was observed. Electroencephalogram improved after five plasma exchange sessions, antithyroglobulin antibody, and thyroid peroxidase antibody, respectively pretreatment of >1,000 IU/ml (normal <4.1 IU/ml), 519 IU/ml (normal for <5.6 IU/ml) dropped to 462 IU/ml, 30 IU/ml. Symptoms of difficulty finding words and understanding speech also improved. In most studies, steroids work within the first 4–6 weeks, while other studies have shown that the time to complete recovery may range from 4 months to 10 years (4). However, in this case, the course of steroids was shorter. If used for a longer duration, the effect may be more pronounced. The patient may be responsive to steroid treatment, but if the clinical symptoms are severe or worsen, plasma exchange can be used to quickly relieve these symptoms. Cook et al. (31) retrospectively analyzed a study on the treatment of Hashimoto's encephalopathy with plasma exchange in 10 cases and showed that 90% of patients had significantly improved symptoms after plasma exchange. Because plasma exchange alone or with other treatments have been used as the second-line treatment for Hashimoto's encephalopathy, in 2016, the American Society for Apheresis published treatment guidelines for plasma exchange and proposed that Hashimoto's encephalopathy is a category II indication of plasma exchange. Moreover, because only observational studies or case series have reported the efficacy of plasma exchange on Hashimoto's encephalopathy and randomized controlled studies are lacking, the recommended level is 2C (2). This study was supported by Simmons and Staley (the volume of plasma exchanged per treatment was 1.0 times the plasma volume) (7) and Endres et al. (8). In addition, Tran et al. (9) found that long-term plasma exchange can be used for maintenance treatment in patients with Hashimoto's encephalopathy accompanied by cerebellar ataxia.

#### Plasma Exchange in Combination With Other Drugs for Hashimoto's Encephalopathy

Plasma exchange is usually combined with steroid, immunoglobulin, and antiepileptic drugs as well as immunosuppressants to treat Hashimoto's encephalopathy (5, 39, 44). Bektas et al. (43) reported a 12-year-old patient with Hashimoto's encephalopathy that mainly manifested status epilepticus, but after the first plasma exchange, his status epilepticus was controlled; subsequently, prednisone combined with a total of nine sessions plasma exchange resulted in normal mental and neurological status in 2 months. Gul et al. (6) also reported two successful cases in which plasma exchange combined with steroid, intravenous immunoglobulin, and antiepileptic drugs to treat Hashimoto's encephalopathy.

However, at present, only case reports and case analyses have shown that plasma exchange is effective at treating Hashimoto's encephalopathy, and because plasma exchange was initiated after failure to respond to steroids or immunoglobulin therapy in most of the studies, we cannot completely rule out the delayed effects of steroids. Nevertheless, because of the narrow time window between clinical symptom improvement and plasma exchange, plasma exchange can be considered for the treatment of Hashimoto's encephalopathy when steroids are ineffective in the short term or when patients cannot tolerate the side effects of steroids, and a few studies have shown that plasma exchange is effective as an initial treatment for Hashimoto's encephalopathy.

#### Side Effects of Plasma Exchange for Hashimoto's Encephalopathy

Plasma exchange is a relatively safe and effective treatment. The application of plasma exchange for the treatment of Hashimoto's encephalopathy is rare, and few studies have evaluated the occurrence of side effects from plasma exchange in Hashimoto's encephalopathy patients. Hussain et al. (44) reported a 54-year-old patient with Hashimoto's encephalopathy treated with plasma exchange who developed a urinary tract infection.

### Application of Plasma Exchange for Limbic Encephalitis

Limbic encephalitis is a neuropsychiatric disease characterized by inflammation of the limbic system, including the hippocampus, amygdala, and less frequently the frontobasal and insular regions. The clinical manifestations are subacute onset of cognitive impairment (mainly short-term memory loss), epilepsy, and mental disorder (46, 47). Radja et al. (48) reported that 97% of VGKC-associated limbic encephalitis presented with memory impairment, 85% with seizures, and 33% with emotional change. Brain MRI may present edema or inflammation that occur selectively on unilateral or bilateral limbic systems, especially in the medial temporal region (49). Electroencephalogram usually shows focal or diffuse slow waves or epileptiform discharge (50), and cerebrospinal fluid usually shows lymphocytosis, slight protein elevation, oligoclonal band positivity, and an increase in the IgG index (51, 52). Limbic encephalitis can be divided into infectious and autoimmune limbic encephalitis according to the etiology. Infectious limbic encephalitis is usually caused by the direct invasion of the brain by pathogens such as herpes simplex virus, while autoimmune limbic encephalitis is caused by an autoimmune disorder and can be divided into paraneoplastic and non-paraneoplastic limbic encephalitis (50).

In 1968, Corsellis et al. (53) used the term “limbic encephalitis” for the first time to describe six patients characterized by progressive memory loss, confusion, and seizures. Of those patients, four had tumors, and three had bronchial carcinomas. An autopsy found that the limbic gray matter in all patients' temporal lobes exhibited inflammation and degeneration, indicating that there was a link between limbic encephalitis and tumors. Gultekin et al. (52) analyzed the relationship between 50 cases of limbic encephalitis and tumors and found that limbic encephalitis commonly occurs with small cell lung cancer (52%, 68/132), testicular cancer (11%, 14/132), and thymoma (5%, 6/132).There were also reports of paraneoplastic limbic encephalitis with non-Hodgkin's lymphoma, neuroblastoma, colon cancer, ovarian cancer, breast cancer, prostate cancer, etc. Since 1988, several studies have confirmed that patients with tumors outside the central nervous system but have neuropsychiatric symptoms have antitumor and brain tissue antibodies in their serum, including anti-Hu, anti-Yo, anti-CRMP5, anti-Ri, anti-Ma2, and anti-amphiphysin antibodies. Since 2000, studies have shown that some limbic encephalitis are detected antibodies against neuronal cell-surface antigens or antibodies against neuronal ion channels, including voltage-gated potassium channels and ligand-gated ion channels and antibodies against VGKC, NMDA, and AMPA receptors, thus providing a therapeutic basis for plasma exchange (47, 50).

#### History of Plasma Exchange for the Treatment of Limbic Encephalitis

Buckley et al. (54) first reported that plasma exchange successfully treated a case of limbic encephalitis. The report described a 47-year-old female stylist with myasthenia gravis. Thymoma was removed 4 years after she was diagnosed with myasthenia gravis, and after her diagnosis of myasthenia gravis for 10 years, her symptoms of myasthenia gravis recurred, the following year, the patient exhibited significant short-term memory loss, irritability, disorientation, inattention, and slowed thinking. The doses of cyclophosphamide and prednisone were reduced, and the patient started using loxapine (fourth generation antipsychotic medication), but her mental status did not improve significantly. After 7 weeks, the patient was transferred to the intensive care unit due to myasthenia crisis, and the myasthenia gravis symptoms were relieved with increased immunosuppression, but her mental status still did not improve. Brain CT and MRI were normal, while cerebrospinal fluid cytology and PCR of herpes simplex virus were negative. The electroencephalogram showed nonspecific slow waves. In the first 10 years after the diagnosis of myasthenia gravis, the VGKC antibody was normal, and significantly increased (750 pM) after the onset of psychiatric symptoms, so the patient was diagnosed with anti-VGKC receptor limbic encephalitis. The myasthenia gravis symptoms and limbic system symptoms improved after six sessions of plasma exchange. Jaben and Winters (55) studied the treatment of five anti-VGKC antibody-related diseases with plasma exchange, of those, four were anti-VGKC antibody-related limbic encephalitis with the main clinical manifestations of memory impairment, seizures, and personality changes. All patients were given 1.0 times the plasma volume during each session every other day for a total of 5–6 sessions. Among these patients, three limbic encephalitis were treated with other immunosuppressive agents at the same time, and one significantly experienced symptom relief with plasma exchange alone. Although there have been a number of cases in which plasma exchange was used to treat limbic encephalitis, plasma exchange in combination with steroid and immunoglobulins have also been used to treat all types of limbic encephalitis. Steroid and immunoglobulin treatments are first-line therapy for limbic encephalitis whereas plasma exchange is not (56). Rather, it is implemented when steroid and immunoglobulin therapy has no obvious effect in the short term, alternatively, plasma exchange may be used in combination with steroid and immunoglobulin to treat limbic encephalitis.

#### Onset Time of Plasma Exchange for Limbic Encephalitis

At present, most studies that describe plasma exchange for limbic encephalitis lack a description of the onset time of plasma exchange. Schimme et al. (57) reported a 12-year-old girl with NMDA receptor encephalitis performed with eight sessions of plasma exchange over 13 days. The patient's clinical symptoms markedly improved after the second plasma exchange, and her ability to walk was partially recovered. This treatment schedule was consistent with the onset time of plasma exchange for anti-NMDA receptor encephalitis that was unresponsive to immunoglobulin (0.4 g/kg/d for 5 days) combined with methylprednisolone (1 g/d for 5 days) in the short term, as reported by Wang et al. (58) in 2015, consciousness was regained after the second plasma exchange. Mazzi et al. (11) reported a case of non-paraneoplastic limbic encephalitis with anti-GAD antibody, in which seizures were significantly reduced by the third plasma exchange. Rypulak et al. (59) reported a 23-year-old patient with NMDA receptor encephalitis who received plasma exchange, and after the third session, the patient's neurological symptoms significantly improved. The GCS score increased to 11 points from 6 points.

#### The Course of Plasma Exchange for Limbic Encephalitis

In the study of Batra et al. (10) and Jaben and Winters (55), the number of plasma exchange sessions used to treat limbic encephalitis ranged from 5 to 6. In 2016, the American Society for Apheresis recommended the use of plasma exchange once every other day to treat anti-NMDA receptor encephalitis, and the volume of plasma exchanged per session was 1–1.5 times the plasma volume. Albumin was used as the replacement fluid, and a total of 5–6 sessions were performed. For the treatment of paraneoplastic neuropathy (PNS), including paraneoplastic limbic encephalitis, plasma exchange is recommended once daily or every other day for a total of 5–6 sessions, and the volume of plasma exchanged per procedure should be 1–1.5 times the plasma volume (2).

#### Clinical Practice of Plasma Exchange for the Treatment of Limbic Encephalitis

Plasma exchange is initiated when steroid and immunoglobulin treatments fail to treat limbic encephalitis, alternatively, it can be combined with steroid and immunoglobulin treatments. Therefore, plasma exchange is usually not the first choice. However, at present, no randomized controlled trials have analyzed the therapeutic effect of plasma exchange on limbic encephalitis. In 2011, Markakis et al. (60) reported a case of a 48-year-old female who presented with mental disorders and disorientation 2 years before admission; the patient rapidly developed anterograde amnesia, irritability, hallucinations, refractory temporal lobe seizure, and obvious short-term memory loss in a few weeks. The enhanced T2 weighted sequence of brain MRI suggested bilateral temporal lobe swelling and revealed a high signal intensity in the medial temporal lobe. High concentrations of anti-GAD antibodies were found in serum and cerebrospinal fluid. A diagnosis of anti-GAD antibody-associated limbic encephalitis was established. After methylprednisolone (1 g/d for 5 days) failed, plasma exchange was initiated, each session involved the replacement of 1.2 times the plasma volume. After a total of seven sessions, the epilepsy was under control, but the patient's cognitive function did not improve. Subsequently, the patient underwent one plasma exchange session every 3 weeks along with oral prednisone (1 mg/kg, gradually reduced to 0.25 mg/kg, for at least 1 year). The seizures did not recur, and a simple intelligence test showed that language and visual memory improved, in addition, anti-GAD antibody levels were reduced at the 1-year follow-up. Mccarthy et al. (61) reported one case of a 32-year-old pregnant woman with anti-NMDA receptor encephalitis, in the first 2 weeks of pregnancy, a new symptom developed, continuous daily headache, and in the maternity clinic, the patient experienced a rapid onset of visual and auditory hallucinations, illusions, irritability, paranoid, delusional within 24 h. One week later, the patient lost consciousness, and seizures began to occur. Brain MRI was normal, EEG showed diffuse slow waves, and cerebrospinal fluid protein was elevated to 726 mg/l (normal is 150–450 mg/l). Steroids were initiated (methyl prednisolone 1 g/d for 5 days, then slowly reduced), but the patient became aggravated, and hence stayed in the intensive care unit. After plasma exchange (1.5 times the plasma volume per session), the patient's symptoms improved significantly, and after 8 weeks, the symptoms of encephalopathy were completely resolved. Van Ael et al. (62) used steroids to treat a 26-year-old woman with GAD antibody limbic encephalitis, but the treatment was unsuccessful. Plasma exchange was subsequently initiated, and marked improvements in clinical symptoms, including memory, seizures, and imaging findings were observed. At the 14-month follow-up, the level of GAD antibody, which was initially >7,000 IU, was decreased by plasma exchange to <1,000 IU. Moreover, several other studies have shown that plasma exchange is more effective than intravenous immunoglobulin and steroids for the treatment of limbic encephalitis, including in pregnant women (10, 12, 61, 63). Korff et al. (64) found that plasma exchange was the most effective at reducing antibody levels in limbic encephalitis.

#### Plasma Exchange in Combination With Other Drugs for the Treatment of Limbic Encephalitis

Plasma exchange is rarely used alone in the treatment of limbic encephalitis, but it is often used in combination with other immunomodulatory therapies such as steroids and/or immunoglobulin. Vincent et al. (65) analyzed the clinical features and treatment of 10 cases of anti-VGKC-antibody-associated limbic encephalitis. Among those cases, seven were treated with plasma exchange combined with steroids or immunoglobulin, and the results showed that four patients experienced a significant curative effect, two patients experienced a slight curative effect, and only one patient did not experience an effect. Desena et al. (66) analyzed the use of plasma exchange to treat 14 cases of anti-NMDA receptor encephalitis, including three adults, 10 patients began plasma exchange after failure to respond to steroids, and the results showed that 7/10 NMDA receptor encephalitis patients who underwent plasma exchange exhibited an average increase in the modified Rankin scale of 0.4, and 3/10 patients exhibited an average increase in the modified Rankin scale of 0.1 after treatment with steroids. The results indicated that plasma exchange combined with steroids was more effective than steroids alone for the treatment of anti-NMDA receptor encephalitis.

However, no randomized controlled trials have been performed to support the therapeutic effect of plasma exchange on limbic encephalitis, and plasma exchange is primarily initiated when the patient exhibits no response to steroid treatment in the short term (perhaps because steroid treatment requires a longer time period to elicit effects). Thus, the therapeutic effect of steroid treatment cannot be completely excluded, especially when it is combined with plasma exchange or immunoglobulin, so improvements in the patient's condition should not be attributed solely to plasma exchange. However, the patients receiving plasma exchange had better outcomes, and plasma exchange exhibited a time-dependent effect. The coincidence was small, and a few cases reported plasma exchange as the initial treatment of Hashimoto's encephalopathy. Therefore, plasma exchange may be considered when patients have contraindications to steroid treatment or slow onset time in the short term, but randomized controlled trials are needed for confirmation.

#### Side Effects of Plasma Exchange for Limbic Encephalitis

Suppiej et al. (67) analyzed the efficacy and side effects of plasma exchange for pediatric NMDA receptor encephalitis and showed two cases of transient hypotension that improved after rehydration therapy and blood vessel vasopressors, one case of allergic reaction and shock because of autonomic dysfunction, and one case of pulmonary embolism. Wong et al. (33) prospectively studied nine VGKC antibody-positive limbic encephalitis patients who underwent five sessions of plasma exchange with 50 ml/kg of plasma volume exchanged per session. Side effects associated with plasma exchange were observed in two patients: one had methicillin-resistant staphylococcus aureus septicemia and vertebral body inflammation, while the other had femoral artery puncture hematoma, deep vein thrombosis, and pulmonary embolism, which improved after treatment. Miyauchi et al. (68) reported an 11-year-old patient with anti-NMDA receptor encephalitis who developed hypotension shock ~1 h after plasma exchange, but the patient's condition improved after rescue. Rypulak et al. (59) reported that hemodynamic instability and coagulation dysfunction occur after treatment of anti-NMDA receptor encephalitis with plasma exchange, and a prolonged interval between plasma exchange sessions can help prevent unwanted side effects. Therefore, the vital signs of patients, especially patients with anti-NMDA receptor encephalitis, should be closely monitored.

### Application of Plasma Exchange in Systemic Lupus Encephalopathy

Systemic lupus erythematosus encephalopathy, also known as neuropsychiatric systemic lupus erythematosus (NPSLE), is a common neurological complication of systemic lupus erythematosus (SLE) (69), patients with NPSLE present a variety of neuropsychological symptoms, including aseptic meningitis, cerebrovascular disease, demyelination, headaches, movement disorders, seizures, confusion, anxiety, cognitive decline, and mood disorders (70), and NPSLE is the main cause of disability and death in patients with systemic lupus erythematosus. The pathogenesis of neuropsychiatric lupus involves a variety of inflammatory factors, autoantibodies, vascular lesions caused by immune complexes, and neuronal dysfunction mediated by autoantibodies (71). For many years, steroids as the first-line drug for systemic lupus erythematosus encephalopathy, often intravenous methylprednisolone (1,000 mg for 3 d), and then oral prednisone (1 mg/kg/d) gradually tapered and stopped within 3–12 months, but long-term use of the steroid has obvious side effects. When SLE encephalopathy has contraindications to steroid, or steroid is not sufficient or ineffective in the short term, plasma exchange may be a rapid-onset, safe, and effective option (72). In 2016, the American Society for Apheresis (ASFA) recommended plasma exchange as a class II indication for severe SLE, including lupus encephalopathy, and the recommended level is 2C (2).

#### History of Plasma Exchange for Systemic Lupus Erythematosus Encephalopathy

In 1976, Jones et al. (71) treated eight SLE patients with plasma exchange and found that plasma exchange can reduce the level of immune complexes in SLE. In 1981, Evans et al. (21) reported a 44-year-old nurse who developed systemic lupus erythematosus encephalopathy 12 years after the diagnosis of SLE, the disease mainly manifests as schizophrenia-like psychosis, personality changes, mood changes, cognitive disorders, delusions, hallucinations, and irrational thoughts and behaviors. High doses of antipsychotic medications such as thiazine, chlorpromazine, and haloperidol combined with prednisone at 200 mg/day failed to treat the patient's mental symptoms. Immune complex levels were significantly increased in the blood and cerebrospinal fluid, and electroencephalograms showed irregular delta waves of 7–8 Hz per s, hence, four sessions of plasma exchange were performed after 35–38 days. An obvious improvement in the patient's mental status was observed 3 days after the fourth plasma exchange. Four weeks later, the dose of prednisone was reduced to 15 mg/d, and all the antipsychotic drugs were stopped at the same time, the patient's mental function and EEG performance gradually returned to normal, and the levels of immune complex and anti-neuronal antibody decreased correspondingly, indicating that plasma exchange can effectively treat lupus encephalopathy in patients with high levels of circulating immune complexes. In 1988, Unterweger et al. (73) reported a case of a 15-year-old girl with lupus encephalopathy. Three years after diagnosis of SLE, the patient manifested a severe depressive episode accompanied by suicidal ideation, disorientation, and delusional behavior, hence, steroids were initiated. After 7 days, the patient still had clinical manifestations of encephalopathy, mainly dementia, and delusions. The symptoms still did not significantly improve within 3 weeks of continued steroids. Therefore, plasma exchange was performed once every 2 days, with a total of five sessions performed over 10 days. The levels of circulating immune complexes decreased, antibody levels decreased, and the symptoms were relieved. Two weeks after the last plasma exchange session, the patient had recovered completely, and based on the results of psychological testing, the patient's attention and memory had returned to normal. Since then, studies have reported that plasma exchange can effectively treat systemic lupus encephalopathy (15, 69, 71, 74, 75). Plasma exchange has recently been reported to successfully treat one lupus encephalopathy case with catatonia as the main manifestation, avoiding the use of electroshock treatment (76). But no randomized controlled trials have confirmed the effect of plasma exchange on systemic lupus erythematosus encephalopathy.

#### The Course of Plasma Exchange for Systemic Lupus Erythematosus Encephalopathy

Kato et al. (77) reported three sessions of plasma exchange for the treatment of systemic lupus erythematosus encephalopathy, and Hussein et al (44) and Perisse et al (76) successfully treated systemic lupus encephalopathy using six sessions. Most studies reporting the use of plasma exchange for lupus encephalopathy lack a specific description of their methods. According to the reports on plasma exchange, for other immune mechanisms mediated neurological diseases, and according to studies reporting the plasma exchange clearance kinetics of immunoglobulin, it should be performed once daily or every other day, with the volume of plasma exchanged per procedure at 1–1.5 times the estimated plasma volume, and a total of 3–6 sessions of plasma exchange.

#### Clinical Practice of Plasma Exchange for the Treatment of Systemic Lupus Erythematosus Encephalopathy

Mild SLE encephalopathy needs only symptomatic treatment, but for severe SLE encephalopathy (disturbance of consciousness, seizures, severe depression, psychotic symptoms, etc.) and lupus encephalopathy in which steroids and cyclophosphamide have contraindications, high concentrations of immune complexes, and steroids have no obvious curative effect in the short term. Plasma exchange can treat lupus encephalopathy effectively, safely, and quickly (78–80). By removing pathogenic autoantibodies, immunoglobulin, immune complexes, and toxins, plasma exchange can also increase the function of regulatory T cells (23, 78). Kato et al. (77) reported a 48-year-old male with lupus encephalopathy that manifested as organic encephalopathy syndrome, specifically exhibiting mental behavior abnormalities, disorientation, memory, and intelligence impairments. Plasma exchange was performed after steroid treatment was deemed ineffective. After the first plasma exchange, the patient's consciousness improved, and their clinical symptoms were relieved. Subsequently, the steroid treatment was continued. Three days after the first plasma exchange session, a second plasma exchange session was conducted, the patient's disorientation was immediately alleviated. Quinter-Del-Rio AI et al. (79) studied a 14-year-old girl with SLE encephalopathy presenting with seizure, after steroid treatment failed, the clinical symptoms were significantly relieved with plasma exchange (1 session/d for 4 days). Gokhale et al. (69) reported three cases of lupus encephalopathy, two of which presented with status epilepticus. Plasma exchange combined with cyclophosphamide was initiated when steroid and cyclophosphamide treatment failed, and the clinical symptoms of all three patients were significantly relieved. Some scholars believe that the combination of synchronous plasma exchange and cyclophosphamide is effective for the treatment of SLE encephalopathy. Neuwelt (32) studied 26 patients who met the 1999 American Society of Rheumatology diagnostic criteria for lupus encephalopathy and treated them with plasma exchange with or without cyclophosphamide. Symptoms were relieved in 74% of patients. Euler et al (80) used plasma exchange and cyclophosphamide to treat 14 cases of severe SLE, of which 12 had complete remission of clinical symptoms within 6 years of follow-up after cessation of treatment.

SLE encephalopathy has various clinical manifestations, and its pathogenesis and pathophysiological mechanisms are still being explored. Although plasma exchange has been shown to be effective at treating SLE encephalopathy, there is a lack of randomized controlled trials to support the efficacy of plasma exchange. Thus, clinicians must weigh the pros and cons of the risk and costs, as well as other factors, to determine whether plasma exchange should be performed. In addition, large-sample, multicenter, randomized controlled trials are still needed to confirm the therapeutic effect of plasma exchange.

#### Side Effects of Plasma Exchange for the Treatment of Systemic Lupus Erythematosus Encephalopathy

Although plasma exchange does have a few side effects, potential life-threatening side effects are very rare, and their incidence is <0.15% (75). Bartolucci et al. (15) analyzed the side effects of plasma exchange as adjuvant treatment for lupus encephalopathy and found five cases: one case of transient hypocalcemia, one case of venous fistula of the indwelling venous catheter, one case of allergic skin reaction to albumin, and two cases of central venous catheter infection.

### Plasma Exchange for the Treatment of ANCA-associated Vasculitis Encephalopathy

ANCA-associated vasculitis includes granulomatosis with polyangiitis (GPA: formerly Wegener's granulomatosis), microscopic polyangiitis (MPA), and eosinophilic granulomatosis with polyangiitis (EGPA: formerly Churg-Strauss syndrome), which is a group of systemic vasculitis characterized by oligovascular oligoimmune necrosis inflammation. ANCAs can be detected in the serum of most patients (81). ANCA-associated vasculitis can lead to systemic organ damage, mainly renal and lung, of which renal damage is more prevalent (70%) (81). However, the manifestations that cause central nervous system damage are not common, with an incidence of ~5–15%. In addition, there is no significant difference in the incidence of each type of ANCA-related vasculitis-induced central nervous system damage. Usually, central nervous system damage is caused by cerebral or spinal vasculitis and granulomas, and the clinical manifestations are complex, ranging from headache to cognitive impairment, memory loss, epilepsy, disturbance of consciousness, paresthesia, etc. (81). Encephalopathy is not a disease, rather, it is a clinical syndrome that describes global brain dysfunction. Mental status alteration is the most typical manifestation, and changes in personality and/or behavior can indicate deterioration in cognitive functioning, a reduction in the level of consciousness, and specific localizing features of injury, such as seizures, ataxia, tremor, and other focal motor signs. There may also be systemic symptoms, such as headache, fever, vomiting, and loss of appetite. In summary, ANCA-associated vasculitis encephalopathy is a global brain dysfunction caused by ANCA-associated vasculitis (82).

Untreated ANCA-associated vasculitis usually leads to death, with a mortality rate as high as 90% in the natural course of 2 years, since the 1950s, when steroids became the basic treatment for ANCA-associated vasculitis, the five-year survival rate has increased to 48%, and after the 1980s, when steroids began to be combined with cyclophosphamide to treat ANCA-associated vasculitis, symptoms could be relieved in 80–90% of patients (83). Because ANCAs play an important role in the pathogenesis of disease, the use of plasma exchange to clear pathogenic antibodies from circulation, and increase the ability of the reticuloendothelial system to clear immune complexes can be combined with immunosuppressive agents for the treatment of severe ANCA-associated vasculitis (84), such as ANCA-associated vasculitis encephalopathy. However, due to the low incidence of ANCA-associated vasculitis encephalopathy, systematic studies on the treatment of ANCA-associated vasculitis encephalopathy by plasma exchange are lacking. There is currently no description of the specific dose and course of plasma exchange for ANCA-associated vasculitis encephalopathy. According to the reports on plasma exchange for other immune mechanisms mediating neurological diseases, it may be recommended once daily or every other day, with the volume of plasma exchanged per session at 1–1.5 times the estimated plasma volume, and a total of five sessions of plasma exchange should be performed.

#### Clinical Practice of Plasma Exchange for the Treatment of ANCA-associated Vasculitis Encephalopathy

The treatment of ANCA-associated vasculitic encephalopathy with plasma exchange is uncommon. In 2000, Deshpande et al. (85) reported a 15-year-old girl with renal failure who developed lethargy for 3 months and suffered from decreased appetite and vomiting in the week prior to admission. At 24 h after admission, renal biopsy showed that 29 of 43 glomeruli contained a mixture of fibrous crescents and cells. The patient was diagnosed with microscopic polyangiitis, and the patient was discharged after her condition improved. Three weeks after discharge, the patient had a severe headache and blurred vision, and blood pressure was 139/92 mmHg. Subsequently, the patient experienced three epileptic seizures, followed by a loss of consciousness, and irritability. Phenytoin and carbamazepine were used to control the seizures, and T2-weighted and T2 FLAIR sequences of brain MRI showed multiple increased signal intensities in the peripheral gray matter of the cerebral hemisphere, especially in the occipital cortex. Vasculitis caused widespread cerebral ischemia. EEG indicated changes consistent with encephalopathy. Headache, disturbance of consciousness, and blurred vision were relieved after the first plasma exchange session, the patient then underwent three sessions of plasma exchange, performed every other day, and after five consecutive plasma exchange sessions, cyclophosphamide was administered intravenously. After 2 months, the abnormal signals on brain MRI showed improvement. Nishio et al (86) reported reversible posterior leukoencephalopathy syndrome caused by vascular damage from Wegener's granulomatosis. A female with Wegener's granulomatosis presented severe headache, nausea, and seizures after severe intestinal complications. Blood pressure was 126/60 mmHg, brain CT showed low-density shadows in the bilateral posterior parietal and occipital lobes, and brain MRI showed high-intensity signals on the T2-weighted sequence in the bilateral occipital white matter, parietal lobe, and frontal lobe. Cerebrospinal fluid was normal. Thus, the diagnosis was reversible posterior white matter encephalopathy syndrome. Two courses of methylprednisolone combined with plasma exchange were administered, followed by intravenous cyclophosphamide at 400 mg/week. After 13 days, brain MRI showed that the abnormal signal had almost completely disappeared. The patient's consciousness and mental status also began to recover, and the concentration of PR3-ANCA antibodies decreased from 164 to 15.7 U/ml, and the intestinal symptoms were relieved. However, studies that treat ANCA-associated vasculitis encephalopathy with plasma exchange are very rare, so more studies are needed in the future to provide supporting evidence of the therapeutic effect of plasma exchange.

### Plasma Exchange for the Treatment of Acute Demyelinating Encephalomyelitis

Acute disseminated encephalomyelitis is an immune-mediated single-phase acute inflammatory demyelinating disease of the central nervous system, which mainly affects the white matter of the brain, brainstem and spinal cord (87). It is common in children and young adults, and most cases occur after viral or bacterial infections or within 2–4 weeks of vaccination (<5%) This disease can exhibit acute or subacute onset, with clinical manifestations of encephalopathy (disturbance of consciousness and behavior change) and multifocal neurological defects. In 2007, an international pediatric multiple sclerosis research group noted that encephalopathy not explained by fever is a necessary clinical manifestation of acute disseminated encephalomyelitis (87, 88), and in the prodromal stage, upper respiratory tract infection, and gastrointestinal symptoms are usually observed. The diagnosis is mainly based on clinical manifestations and imaging examination. Typical cerebrospinal fluid changes include increased pressure, increased lymphocytes, increased protein (usually <1.0 mg/l), and normal glucose. The gamma globulin and IgG levels in cerebrospinal fluid can increase, as can myelin basic protein, with a rare oligoclonal band. Electroencephalography examination presents a slow wave on imaging and is of great value in the diagnosis of acute disseminated encephalomyelitis, especially brain MRI, which can detect multiple or extensive white matter or deep gray matter lesions (thalamus and basal ganglia) within 5–14 days after the onset of symptoms (88, 89).

The pathogenesis of acute disseminated encephalomyelitis is not very clear. Cell-mediated immune dysfunction may play a main role. Humoral factors, including antibodies, complement, immune complex, and cytokines, also play an important role (78), so priority is given to immune modulators (90). No randomized controlled trials have been performed on children or adults to determine the best treatment for ADEM. Systemic intravenous large doses of corticoids are currently considered to be first-line therapy (89). Early intravenous methylprednisolone can shorten the course of the disease. Plasma exchange has been reported to be effective in the treatment of acute disseminated encephalomyelitis when the diagnosis of acute disseminated encephalomyelitis is delayed and cannot be early except for infectious diseases where steroid cannot be used, where there are contraindications to steroid, or where the effects of steroid are not obvious in the short term (90).

#### History of Plasma Exchange for the Treatment of Acute Disseminated Encephalomyelitis

In 1981, Newton et al. successfully treated the first case of acute disseminated encephalomyelitis caused by acute infection through plasma exchange alone (4). Subsequently, Cotter et al. (91) reported the case of a 22-year-old male with acute disseminated encephalomyelitis caused by mycoplasma pneumoniae infection. Eight days after onset, the patient improved after plasma exchange alone. Several studies have shown that plasma exchange can effectively treat acute disseminated encephalomyelitis. However, the current therapeutic effect of plasma exchange lacks the support of randomized controlled trials.

#### Onset Time of Plasma Exchange for the Treatment of Acute Disseminated Encephalomyelitis

Plasma exchange for the treatment of acute disseminated encephalomyelitis usually takes effect after the first plasma exchange (88). Kanter et al. (92) reported that plasma exchange for the treatment of two cases of acute disseminated encephalomyelitis, in which one improved neurological function within a few hours during the first plasma exchange. Shah et al. (93) noted, in their study of plasma exchange for the treatment of one acute disseminated encephalomyelitis, that the neurological deficit was improved after the first plasma exchange, which was supported by findings reported in the study by Yi et al. (94).

#### Course of Plasma Exchange for the Treatment of Acute Disseminated Encephalomyelitis

The course of plasma exchange for the treatment of acute disseminated encephalomyelitis has not been determined. Borras-Novell et al. (90) and Stricker et al. (95), in the study of plasma exchange for treating acute fulminant disseminated encephalomyelitis, included four cases of acute disseminated encephalomyelitis. The number of plasma exchange sessions was 3–10, predominantly 5, and plasma exchange was performed once daily with the volume of plasma exchanged per procedure set at one times the plasma volume, with 5% albumin used as replacement fluid, and all patients improved. Therefore, for the treatment of acute disseminated encephalomyelitis, 3–10 sessions are recommended, typically five, performed once a day or every other day, with the volume of plasma exchanged per sessions set at one times the plasma volume and albumin used as the replacement fluid.

#### Clinical Practice of Plasma Exchange in Acute Disseminated Encephalomyelitis

Miyazawa et al. (96) reported an eleven-year-old patient with acute disseminated encephalomyelitis. Brain MRI revealed extensive multiple high-intensity lesions in the white matter on T2-weighted imaging. Intravenous immunoglobulin (0.125 g/kg/d for 3 days) was administered after 10 days of illness, and 11 days after onset, oral prednisone was initiated at a dose of 60 mg/d, but the patient did not improve. Twelve days after onset, intravenous methylprednisolone was administered (1,000 mg/d) for 3 days. Fourteen days after onset, the patient became comatose, and spinal magnetic resonance T2 weighted images showed cervical segmental spinal cord swelling and abnormally high signal because of the patients' neurologic deterioration. At 17–20 days after onset, three plasma exchange sessions were started using 5% albumin as the replacement fluid. She regained full consciousness, and MRI improved. Lin et al. (97) described plasma exchange for the successful treatment of two cases of acute disseminated encephalomyelitis, including a 26-year-old woman who had a history of rubella vaccination, characterized by disturbance of consciousness, speech, ataxia, and myoclonus. Brain magnetic resonance T2 weighted images revealed high-intensity signals in the brain stem, cerebellum, basal ganglia, thalamus, and white matter of the cerebral ventricles. Initially, she received intravenous methylprednisolone (1,000 mg/d for 5 days and then 500 mg/d for 2 days) and then oral prednisone. However, her neurological function began to deteriorate, and one seizure occurred after 3 days of intravenous methylprednisolone. Five sessions of plasma exchange were initiated, with one session performed every 2 days, and 5% of albumin was used as the replacement fluid. Her symptoms were alleviated after the third plasma exchange. Another a 65-year-old patient presented with acute disseminated encephalomyelitis, unconsciousness, speech disorder, emotional dissonance, and slow movement. T2-weighted images of the brain magnetic resonance showed high-intensity signals in the bilateral cerebral hemisphere, an elevated number of cells in the cerebrospinal fluid, normal glucose, and moderately elevated protein levels. She received five plasma exchange sessions (once every 2 days, with 5% albumin as the replacement fluid). After the fourth plasma exchange, her symptoms began to resolve.

Since most studies have shown that the standard dose of immunoglobulin for the treatment of acute disseminated encephalomyelitis is 0.4 g/kg/d for 5 days, steroids are usually administered intravenously at a dose of 20–30 mg/kg/d for (3–5 days), followed by oral prednisone at a dose of 1–2 mg/kg/d (87). Miyazawa and Lin et al reported that a poor response to intravenous immunoglobulin and steroids may be related to inadequate immunoglobulin and steroid dosing. Even so, the delayed effects of steroid and immunoglobulin treatments cannot be excluded as a component of symptom resolution because of the narrow time window between symptom improvement and plasma exchange, indicating that plasma exchange can effectively treat fulminant acute disseminated encephalomyelitis. In addition, Stricker et al. (95) reported four cases of acute disseminated encephalomyelitis due to delayed diagnosis and cannot exclude infection, which were significantly improved by plasma exchange alone. Keegan et al. (98) reviewed 59 cases of acute and severe central nervous system demyelination treated with plasma exchange at the Mayo clinic, including 10 patients with acute disseminated encephalomyelitis (ADEM), of whom 40% experienced symptom relief. Therefore, plasma exchange treatment for acute disseminated encephalomyelitis is effective, and few side effects have been reported. However, there is still a lack of support from randomized controlled studies.

Other types of steroid-responsive encephalopathy are very rarely reported, including hypoglycemic encephalopathy (99) and steroid-responsive encephalopathy caused by cholesterol thrombosis (100), and currently, there are no reports on the effects of plasma exchange therapy on these types.

## Conclusion

In conclusion, plasma exchange is a widely accepted treatment method for autoimmune neurological diseases because it is safe, rapid-onset, and effective. Plasma exchange may be effective for patients with steroid-responsive encephalopathy who do not respond to treatment with steroids in the short term or those who have contraindications for steroid treatment, including patients with Hashimoto's encephalopathy, limbic encephalitis, SLE encephalopathy, ANCA-associated vasculitis encephalopathy, or acute disseminated encephalomyelitis. The main limitation of this study is the lack of systematic treatments reported in the literature. The current data are almost entirely derived from case reports and case analyses. Moreover, plasma exchange is often applied in combination with steroids and other immunosuppressive agents, hence, no high-quality evidence is available to prove the efficacy of plasma exchange on steroid-responsive encephalopathy.

## Author Contributions

YJ, XT, and XW provided the initial idea and outline of content for the manuscript. All authors contributed content and critically reviewed and edited the manuscript.

### Conflict of Interest Statement

The authors declare that the research was conducted in the absence of any commercial or financial relationships that could be construed as a potential conflict of interest.

## References

[1] Schwartz J. Evidence-based guideline update: plasmapheresis in neurologic disorders. Neurology. (2011) 77:e105–6; author reply: e6. 10.1212/WNL.0b013e318230b33f 22135818

[2] Schwartz J Padmanabhan A Aqui N Balogun RA Connelly-Smith L Delaney M. Guidelines on the use of therapeutic apheresis in clinical practice-evidence-based approach from the writing committee of the american society for apheresis: the seventh special issue. J Clin Apheresis. (2016) 31:149–62. 10.1002/jca.21470 27322218

[3] Castillo P Woodruff B Caselli R Vernino S Lucchinetti C Swanson J. Steroid-responsive encephalopathy associated with autoimmune thyroiditis. Arch Neurol. (2006) 63:197–202. 10.1001/archneur.63.2.197 16476807

[4] Mocellin R Walterfang M Velakoulis D. Hashimoto's encephalopathy: epidemiology, pathogenesis and management. CNS Drugs. (2007) 21:799–811. 10.2165/00023210-200721100-00002 17850170

[5] Nagpal T Pande S. Hashimoto's encephalopathy: response to plasma exchange. Neurol. India. (2004) 52:245–7. 15269483

[6] Gul Mert G Horoz OO Herguner MO Incecik F Yildizdas RD Onenli Mungan N. Hashimoto's encephalopathy: four cases and review of literature. Int J Neurosci. (2014) 124:302–6. 10.3109/00207454.2013.836706 23967879

[7] Simmons SC Staley EM Dorn DP Nails AP Marques MB Pham HP. Therapeutic plasma exchange For Hashimoto's encephalopathy. J Clin Apher. (2018) 33:444–6. 10.1002/jca.21597 29083097

[8] Endres D Vry MS Dykierek P Riering AN Lungen E Stich O. Plasmapheresis responsive rapid onset dementia with predominantly frontal dysfunction in the context of Hashimoto's encephalopathy. Front Psychiatry. (2017) 8:212. 10.3389/fpsyt.2017.00212 29123489 PMC5662557

[9] Tran MH Mkhikian H Sy M Perez-Alvarez I Demetriou M. Long-term plasma exchange as maintenance therapy for cerebellar-type Hashimoto's encephalopathy, a case report. Transfus Apher Sci. (2018) 57:418–20. 10.1016/j.transci.2018.05.027 29891220

[10] Batra R Pang Y Friedman MT. Therapeutic plasma exchange in anti-N-methyl-D-aspartate-receptor (anti-NMDA-R) encephalitis associated with benign ovarian teratoma. J Clin Apher. (2012) 27:227–8. 10.1002/jca.21212 22411418

[11] Mazzi G Roia DD Cruciatti B Mata S Catapano R. Plasma exchange for anti-GAD associated non paraneoplastic limbic encephalitis. Transfus Apher Sci. (2008) 39:229–33. 10.1016/j.transci.2008.09.005 18955013

[12] Martin IW Martin CL Dunbar NM Lee SL Szczepiorkowski ZM. Therapeutic plasma exchange as a steroid-sparing therapy in a patient with limbic encephalitis due to antibodies to voltage-gated potassium channels. J Clin Apher. (2016) 31:63–5. 10.1002/jca.21395 25851808

[13] Ozkale M Erol I Ozkale Y Kozanoglu I Overview of therapeutic plasma exchange in pediatric neurology: a single-center experience. Acta Neurol Belgica. (2018) 118:451–8. 10.1007/s13760-018-0961-5 29882008

[14] Hussein M Mooij J Roujouleh H. Cerebral lupus in patients whilst on treatment for lupus nephritis with cyclosporine. J Clin Neurosci. (2003) 10:104–6. 10.1016/S0967-5868(02)00260-6 12464536

[15] Bartolucci P Brechignac S Cohen P Le Guern V Guillevin L. Adjunctive plasma exchanges to treat neuropsychiatric lupus: a retrospective study on 10 patients. Lupus. (2007) 16:817–22. 10.1177/0961203307081840 17895305

[16] Shinozaki K Oda S Sadahiro T Nakamura M Abe R Nakamura S. A case report of plasmapheresis in the treatment of acute disseminated encephalomyelitis. Ther Apher Dial. (2008) 12:401–5. 10.1111/j.1744-9987.2008.00617.x 18937725

[17] Balestri P Grosso S Acquaviva A Bernini M. Plasmapheresis in a child affected by acute disseminated encephalomyelitis. Brain Dev. (2000) 22:123–6. 10.1016/S0387-7604(99)00115-1 10722965

[18] Abel JJ Rowntree LG Turner BB. Plasma removal with return of corpuscles (plasmaphaeresis). The journal of pharmacology and experimental therapeutics Vol. V. No. 6, July, 1914. Transfusion Sci. (1990) 11:166–77. 10160881

[19] Adams WS Blahd WH Bassett SH. A method of human plasmapheresis. Proc Soc Exp Biol Med. (1952) 80:377–9. 10.3181/00379727-80-19629 14949057

[20] Schwab PJ Fahey JL. Treatment of Waldenstrom's macroglobulinemia by plasmapheresis. N Engl J Med. (1960) 263:574–9. 10.1056/NEJM196009222631202 14443924

[21] Evans DT Giles M Horne DJ d'Apice AJ Riglar A Toh BH. Cerebral lupus erythematosus responding to plasmaphaeresis. Postgraduate Med J. (1981) 57:247–51. 10.1136/pgmj.57.666.247 7291106 PMC2424992

[22] Popescu A Kao AH. Neuropsychiatric systemic lupus erythematosus. Curr Neuropharmacol. (2011) 9:449–57. 10.2174/157015911796557984 22379459 PMC3151599

[23] Winters JL. Plasma exchange: concepts, mechanisms, and an overview of the American Society for Apheresis guidelines. Hematol Am Soc Hematol Educ Program. (2012) 2012:7–12. 10.1182/asheducation-2012.1.7 23233554

[24] Dau PC. Increased antibody production in peripheral blood mononuclear cells after plasma exchange therapy in multiple sclerosis. J Neuroimmunol. (1995) 62:197–200. 10.1016/0165-5728(95)00121-4 7499508

[25] Steven MM Tanner AR Holdstock GE Cockerell R Smith J Smith DS. The effect of plasma exchange on the *in vitro* monocyte function of patients with immune complex diseases. Clin Exp Immunol. (1981) 45:240–5. 6459197 PMC1537382

[26] Tesar V Jelinkova E Masek Z Jirsa M Jr Zabka J Bartunkova J. Influence of plasma exchange on serum levels of cytokines and adhesion molecules in ANCA-positive renal vasculitis. Blood Purif. (1998) 16:72–80. 10.1159/000014316 9572400

[27] Yeh JH Wang SH Chien PJ Shih CM Chiu HC. Changes in serum cytokine levels during plasmapheresis in patients with myasthenia gravis. Eur J Neurol. (2009) 16:1318–22. 10.1111/j.1468-1331.2009.02729.x 19614971

[28] Natarajan N Weinstein R. Therapeutic apheresis in neurology critical care. J Intensive Care Med. (2005) 20:212–25. 10.1177/0885066605276816 16061904

[29] Guyatt GH Oxman AD Vist GE Kunz R Falck-Ytter Y Alonso-Coello P. GRADE: an emerging consensus on rating quality of evidence and strength of recommendations. BMJ (2008) 336:924–6. 10.1136/bmj.39489.470347.AD 18436948 PMC2335261

[30] Kaplan AA. Therapeutic plasma exchange: a technical and operational review. J Clin Apheresis. (2013) 28:3–10. 10.1002/jca.21257 23420589

[31] Cook MK Malkin M Karafin MS. The use of plasma exchange in Hashimoto's encephalopathy: a case report and review of the literature. J Clin Apheresis (2015) 30:188–92. 10.1002/jca.21353 25116126

[32] Neuwelt CM. The role of plasmapheresis in the treatment of severe central nervous system neuropsychiatric systemic lupus erythematosus. Ther Apher Dial. (2003) 7:173–82. 10.1046/j.1526-0968.2003.00032.x 12918940

[33] Wong SH Saunders MD Larner AJ Das K Hart IK. An effective immunotherapy regimen for VGKC antibody-positive limbic encephalitis. J Neurol Neurosurg Psychiatry. (2010) 81:1167–9. 10.1136/jnnp.2009.178293 20660916

[34] Basic-Jukic N Kes P Glavas-Boras S Brunetta B Bubic-Filipi L Puretic Z. Complications of therapeutic plasma exchange: experience with 4857 treatments. Ther Apher Dial. (2005) 9:391–5. 10.1111/j.1744-9987.2005.00319.x 16202013

[35] Kaynar L Altuntas F Aydogdu I Turgut B Kocyigit I Hacioglu SK. Therapeutic plasma exchange in patients with neurologic diseases: retrospective multicenter study. Transfusion Apher Sci. (2008) 38:109–15. 10.1016/j.transci.2007.11.002 18331814

[36] Casian A Jayne D. Plasma exchange in the treatment of Wegener's granulomatosis, microscopic polyangiitis, Churg-Strauss syndrome and renal limited vasculitis. Curr Opin Rheumatol. (2011) 23:12–7. 10.1097/BOR.0b013e32834120c1 21124082

[37] Brain L Jellinek EH Ball K. Hashimoto's disease and encephalopathy. Lancet. (1966) 2:512–4. 10.1016/S0140-6736(66)92876-5 4161638

[38] Riangwiwat T Sangtian J Sriphrapradang C. Steroid-responsive encephalopathy: an under recognised aspect of Hashimoto's thyroiditis. BMJ Case Rep. (2015) 2015:bcr2014208969. 10.1136/bcr-2014-208969 25766444 PMC4368923

[39] Chong JY Rowland LP Utiger RD Hashimoto encephalopathy: syndrome or myth? Arch Neurol. (2003) 60:164–71. 10.1001/archneur.60.2.164 12580699

[40] Pari E Rinaldi F Premi E Codella M Rao R Paghera B A follow-up (1)(8)F-FDG brain PET study in a case of Hashimoto's encephalopathy causing drug-resistant status epilepticus treated with plasmapheresis. J Neurol. (2014) 261:663–7. 10.1007/s00415-013-7228-0 24390201

[41] Laurent C Capron J Quillerou B Thomas G Alamowitch S Fain O. Steroid-responsive encephalopathy associated with autoimmune thyroiditis (SREAT): characteristics, treatment and outcome in 251 cases from the literature. Autoimmun Rev. (2016) 15:1129–33. 10.1016/j.autrev.2016.09.008 27639840

[42] Boers PM Colebatch JG. Hashimoto's encephalopathy responding to plasmapheresis. J Neurol Neurosurg Psychiatry. (2001) 70:132. 10.1136/jnnp.70.1.132 11118266 PMC1763442

[43] Bektas O Yilmaz A Kendirli T Siklar Z Deda G. Hashimoto encephalopathy causing drug-resistant status epilepticus treated with plasmapheresis. Pediatr Neurol. (2012) 46:132–5. 10.1016/j.pediatrneurol.2011.11.009 22264710

[44] Hussain NS Rumbaugh J Kerr D Nath A Hillis AE. Effects of prednisone and plasma exchange on cognitive impairment in Hashimoto encephalopathy. Neurology. (2005) 64:165–6. 10.1212/01.WNL.0000148580.98997.C5 15642930

[45] Nieuwenhuis L Santens P Vanwalleghem P Boon P. Subacute Hashimoto's encephalopathy, treated with plasmapheresis. Acta Neurolog Belgica. (2004) 104:80–3. 15508271

[46] Mori M Kuwabara S Yoshiyama M Kanesaka T Ogata T Hattori T. Successful immune treatment for non-paraneoplastic limbic encephalitis. J Neurol Sci. (2002) 201:85–8. 10.1016/S0022-510X(02)00188-0 12163199

[47] Honnorat J. Autoimmune limbic encephalitis: an expanding concept. Lancet Neurol. (2010) 9:24–5. 10.1016/S1474-4422(09)70333-3 19962349

[48] Radja GK Cavanna AE. Treatment of VGKC complex antibody-associated limbic encephalitis: a systematic review. J Neuropsychiatry Clin Neurosci. (2013) 25:264–71. 10.1176/appi.neuropsych.13020022 24247853

[49] Dirr LY Elster AD Donofrio PD Smith M. Evolution of brain MRI abnormalities in limbic encephalitis. Neurology. (1990) 40:1304–6. 10.1212/WNL.40.8.1304 2166250

[50] Vedeler CA Storstein A Autoimmune limbic encephalitis. Acta Neurol Scand Suppl. (2009) 120:63–7. 10.1111/j.1600-0404.2009.01204.x 19566502

[51] Lawn ND Westmoreland BF Kiely MJ Lennon VA Vernino S. Clinical, magnetic resonance imaging, and electroencephalographic findings in paraneoplastic limbic encephalitis. Mayo Clin Proceed. (2003) 78:1363–8. 10.4065/78.11.1363 14601695

[52] Gultekin SH Rosenfeld MR Voltz R Eichen J Posner JB Dalmau J. Paraneoplastic limbic encephalitis: neurological symptoms, immunological findings and tumour association in 50 patients. Brain. (2000) 123 (Pt 7):1481–94. 10.1093/brain/123.7.1481 10869059

[53] Corsellis JA Goldberg GJ Norton AR. “Limbic encephalitis” and its association with carcinoma. Brain. (1968) 91:481–96. 10.1093/brain/91.3.481 5723018

[54] Buckley C Oger J Clover L Tuzun E Carpenter K Jackson M. Potassium channel antibodies in two patients with reversible limbic encephalitis. Ann Neurol. (2001) 50:73–8. 10.1002/ana.1097 11456313

[55] Jaben EA Winters JL. Plasma exchange as a therapeutic option in patients with neurologic symptoms due to antibodies to voltage-gated potassium channels: a report of five cases and review of the literature. J Clin Apher. (2012) 27:267–73. 10.1002/jca.21233 22532193

[56] Gastaldi M Thouin A Vincent A. Antibody-mediated autoimmune encephalopathies and immunotherapies. Neurotherapeutics. (2016) 13:147–62. 10.1007/s13311-015-0410-6 26692392 PMC4720680

[57] Schimmel M Bien CG Vincent A Schenk W Penzien J. Successful treatment of anti-N-methyl-D-aspartate receptor encephalitis presenting with catatonia. Arch Dis Childhood. (2009) 94:314–6. 10.1136/adc.2008.149021 19307202

[58] Wang RJ Chen BD Qi D. Anti-N-methyl-D-aspartate receptor encephalitis concomitant with multifocal subcortical white matter lesions on magnetic resonance imaging: a case report and review of the literature. BMC Neurol. (2015) 15:107. 10.1186/s12883-015-0366-5 26152327 PMC4495850

[59] Rypulak E Borys M Piwowarczyk P Fijalkowska M Potrec B Sysiak J. Successful treatment of anti-NMDA receptor encephalitis with a prompt ovarian tumour removal and prolonged course of plasmapheresis: a case report. Mol Clin Oncol. (2016) 5:845–9. 10.3892/mco.2016.1054 28101360 PMC5228193

[60] Markakis I Alexopoulos H Poulopoulou C Akrivou S Papathanasiou A Katsiva V. Immunotherapy-responsive limbic encephalitis with antibodies to glutamic acid decarboxylase. J Neurol Sci. (2014) 343:192–4. 10.1016/j.jns.2014.05.032 24950904

[61] McCarthy A Dineen J McKenna P Keogan M Sheehan J Lynch T. Anti-NMDA receptor encephalitis with associated catatonia during pregnancy. J Neurol. (2012) 259:2632–5. 10.1007/s00415-012-6561-z 22752087

[62] Van Ael Y Amir R Cras P. Anti-GAD antibodies, a rare cause of limbic encephalitis: a case report. Acta Neurol Belgica. (2016) 116:105–7. 10.1007/s13760-015-0493-1 26032353

[63] Shahani L. Steroid unresponsive anti-NMDA receptor encephalitis during pregnancy successfully treated with plasmapheresis. BMJ Case Rep. (2015) 2015:bcr2014208823. 10.1136/bcr-2014-208823 25926583 PMC4422904

[64] Korff CM Parvex P Cimasoni L Wilhelm-Bals A Hampe CS Schwitzgebel VM. Encephalitis associated with glutamic acid decarboxylase autoantibodies in a child: a treatable condition? Arch Neurol. (2011) 68:1065–8. 10.1001/archneurol.2011.177 21825244

[65] Vincent A Buckley C Schott JM Baker I Dewar BK Detert N. Potassium channel antibody-associated encephalopathy: a potentially immunotherapy-responsive form of limbic encephalitis. Brain. (2004) 127 (Pt 3):701–12. 10.1093/brain/awh077 14960497

[66] DeSena AD Noland DK Matevosyan K King K Phillips L Qureshi SS. Intravenous methylprednisolone versus therapeutic plasma exchange for treatment of anti-N-methyl-D-aspartate receptor antibody encephalitis: a retrospective reiview. J Clin Apher. (2015) 30:212–6. 10.1002/jca.21363 25664728

[67] Suppiej A Nosadini M Zuliani L Pelizza MF Toldo I Bertossi C. Plasma exchange in pediatric anti-NMDAR encephalitis: a systematic review. Brain Dev. (2016) 38:613–22. 10.1016/j.braindev.2016.01.009 26926399

[68] Miyauchi A Monden Y Osaka H Takahashi Y Yamagata T. A case of anti-NMDAR encephalitis presented hypotensive shock during plasma exchange. Brain Dev. (2016) 38:427–30. 10.1016/j.braindev.2015.10.009 26524986

[69] Gokhale YA Bhide S Rajadhyaksha S Bichile LS Hase NK. Plasmapheresis: an adjunct therapy in severe progressive neuropsychiatric lupus. J Assoc Phys India. (2001) 49:986–9. 11848331

[70] The American College of Rheumatology nomenclature and case definitions for neuropsychiatric lupus syndromes Arthritis Rheum. (1999) 42:599–608. 10.1002/1529-0131(199904)42:4<599::AID-ANR2>3.0.CO;2-F 10211873

[71] Jones JV Cumming RH Bucknall RC Asplin CM. Plasmapheresis in the management of acute systemic lupus erythematosus? Lancet. (1976) 1:709–11. 10.1016/S0140-6736(76)93088-9 56531

[72] Magro-Checa C Zirkzee EJ Huizinga TW Steup-Beekman GM. Management of neuropsychiatric systemic lupus erythematosus: current approaches and future perspectives. Drugs. (2016) 76:459–83. 10.1007/s40265-015-0534-3 26809245 PMC4791452

[73] Unterweger B Klein G Fleischhacker WW. Plasma exchange for cerebral lupus erythematosus. Biol Psychiatry. (1988) 24:946–7. 10.1016/0006-3223(88)90233-8 3233235

[74] Barath S Soltesz P Kiss E Aleksza M Zeher M Szegedi G. The severity of systemic lupus erythematosus negatively correlates with the increasing number of CD4+CD25(high)FoxP3+ regulatory T cells during repeated plasmapheresis treatments of patients. Autoimmunity. (2007) 40:521–8. 10.1080/08916930701610028 17966042

[75] Cid J Carbasse G Andreu B Baltanas A Garcia-Carulla A Lozano M. Efficacy and safety of plasma exchange: ann 11-year single-center experience of 2730 procedures in 317 patients. Transfus Apher Sci. (2014) 51:209–14. 10.1016/j.transci.2014.08.018 25217991

[76] Perisse D Amoura Z Cohen D Saintigny P Mekhloufi F Mazet P. Case study: effectiveness of plasma exchange in an adolescent with systemic lupus erythematosus and catatonia. J Am Acad Child Adoles Psychiatry. (2003) 42:497–9. 10.1097/01.CHI.0000046820.95464.86 12649638

[77] Kato T Shiratori K Kobashigawa T Hidaka Y. Systemic lupus erythematosus with organic brain syndrome: serial electroencephalograms accurately evaluate therapeutic efficacy. Internal Med. (2006) 45:95–9. 10.2169/internalmedicine.45.1363 16484747

[78] Lassmann H Brunner C Bradl M Linington C. Experimental allergic encephalomyelitis: the balance between encephalitogenic T lymphocytes and demyelinating antibodies determines size and structure of demyelinated lesions. Acta Neuropathol. (1988) 75:566–76. 10.1007/BF00686201 3259787

[79] Quintero-Del-Rio AI Van M. Neurologic symptoms in children with systemic lupus erythematosus. J Child Neurol. (2000) 15:803–7. 10.1177/088307380001501207 11198495

[80] Euler HH Schroeder JO Harten P Zeuner RA Gutschmidt HJ. Treatment-free remission in severe systemic lupus erythematosus following synchronization of plasmapheresis with subsequent pulse cyclophosphamide. Arthritis Rheum. (1994) 37:1784–94. 10.1002/art.1780371212 7986225

[81] Lally L Spiera R. Current therapies for ANCA-associated vasculitis. Annu Rev Med. (2015) 66:227–40. 10.1146/annurev-med-011514-023051 25341007

[82] Davies E Connolly DJ Mordekar SR. Encephalopathy in children: an approach to assessment and management. Arch Dis Childhood. (2012) 97:452–8. 10.1136/adc.2011.300998 22201646

[83] Rubio-Agusti I Salavert M Bataller L. Limbic encephalitis and related cortical syndromes. Curr Treat Options Neurol. (2013) 15:169–84. 10.1007/s11940-012-0212-7 23250843

[84] Ribi C Cohen P Pagnoux C Mahr A Arene JP Puechal X. Treatment of polyarteritis nodosa and microscopic polyangiitis without poor-prognosis factors: a prospective randomized study of one hundred twenty-four patients. Arthritis Rheum. (2010) 62:1186–97. 10.1002/art.27340 20131268

[85] Deshpande PV Gilbert R Alton H Milford DV. Microscopic polyarteritis with renal and cerebral involvement. Pediatr Nephrol. (2000) 15:134–5. 10.1007/s004670000428 11095031

[86] Nishio M Yoshioka K Yamagami K Morikawa T Konishi Y Hayashi N. Reversible posterior leukoencephalopathy syndrome: a possible manifestation of Wegener's granulomatosis-mediated endothelial injury. Modern Rheumatol. (2008) 18:309–14. 10.3109/s10165-008-0052-1 18415039

[87] Pohl D Tenembaum S. Treatment of acute disseminated encephalomyelitis. Curr Treat Options Neurol. (2012) 14:264–75. 10.1007/s11940-012-0170-0 22476745

[88] Krupp LB Tardieu M Amato MP Banwell B Chitnis T Dale RC. International pediatric multiple sclerosis study group criteria for pediatric multiple sclerosis and immune-mediated central nervous system demyelinating disorders: revisions to the 2007 definitions. Multiple Scler. (2013) 19:1261–7. 10.1177/1352458513484547 23572237

[89] Gray MP Gorelick MH. Acute disseminated encephalomyelitis. Pediatr Emergency Care. (2016) 32:395–400. 10.1097/PEC.0000000000000825 27253358

[90] Borras-Novell C Garcia Rey E Perez Baena LF Jordan Garcia I Catella Cahiz D Cambra F. Therapeutic plasma exchange in acute disseminated encephalomyelitis in children. J Clin Apher. (2015) 30:335–9. 10.1002/jca.21388 26332469

[91] Cotter FE Bainbridge D Newland AC. Neurological deficit associated with *Mycoplasma pneumoniae* reversed by plasma exchange. Br Med J. (1983) 286:22. 10.1136/bmj.286.6358.22 6401445 PMC1546620

[92] Kanter DS Horensky D Sperling RA Kaplan JD Malachowski ME Churchill WH Jr. Plasmapheresis in fulminant acute disseminated encephalomyelitis. Neurology. (1995) 45:824–7. 10.1212/WNL.45.4.824 7723979

[93] Shah AK Tselis A Mason B. Acute disseminated encephalomyelitis in a pregnant woman successfully treated with plasmapheresis. J Neurol Sci. (2000) 174:147–51. 10.1016/S0022-510X(00)00260-4 10727701

[94] Yi CH La Vega-Talbott M Friedman MT. Treatment of acute disseminated encephalomyelitis with plasmapheresis in a 16-year-old female, a case report and literature review. J Clin Apher. (2014) 29:339–40. 10.1002/jca.21327 24824150

[95] Stricker RB Miller RG Kiprov DD. Role of plasmapheresis in acute disseminated (postinfectious) encephalomyelitis. J Clin Apher. (1992) 7:173–9. 10.1002/jca.2920070403 1299654

[96] Miyazawa R Hikima A Takano Y Arakawa H Tomomasa T Morikawa A. Plasmapheresis in fulminant acute disseminated encephalomyelitis. Brain Dev. (2001) 23:424–6. 10.1016/S0387-7604(01)00256-X 11578855

[97] Lin CH Jeng JS Yip PK. Plasmapheresis in acute disseminated encephalomyelitis. J Clin Apher. (2004) 19:154–9. 10.1002/jca.20022 15493052

[98] Keegan M Pineda AA McClelland RL Darby CH Rodriguez M Weinshenker BG. Plasma exchange for severe attacks of CNS demyelination: predictors of response. Neurology. (2002) 58:143–6. 10.1212/WNL.58.1.143 11781423

[99] Mathur JL Rajabi F Schroeder A Becker TK. Case of steroid-responsive encephalopathy from hypoglycaemia. BMJ Case Rep. (2017) 2017:bcr-2017-221262. 10.1136/bcr-2017-221262 28838925 PMC5623988

[100] Laloux P. Steroid-responsive leucoencephalopathy due to cholesterol embolism. J Neurol Neurosurg Psychiatry. (2007) 78:112. 10.1136/jnnp.2006.105981 17229741 PMC2077677

